# An alternative application of Rasch analysis to assess data from ophthalmic patient-reported outcome instruments

**DOI:** 10.1371/journal.pone.0197503

**Published:** 2018-06-21

**Authors:** Richard N. McNeely, Salissou Moutari, Samuel Arba-Mosquera, Shwetabh Verma, Jonathan E. Moore

**Affiliations:** 1 Cathedral Eye Clinic, 89-91 Academy Street, Belfast BT1 2 LS, Northern Ireland, United Kingdom; 2 Biomedical Sciences Research Institute, University of Ulster, Coleraine, Northern Ireland, United Kingdom; 3 School of Mathematics and Physics, Queens University Belfast, University Road, Belfast BT7 1NN, Northern Ireland, United Kingdom; 4 Biomedical Engineering Office, Research and Development, SCHWIND Eye-Tech-Solutions, Mainparkstraße 6-10, D-63801 Kleinostheim, Germany; 5 Experimental Radiation Oncology, University Medical Center Mannheim, Heidelberg University, Heidelberg, Germany; 6 Interdisciplinary Center for Scientific Computing (IWR), Heidelberg University, Heidelberg, Germany; 7 Central Institute for Computer Engineering (ZITI), Heidelberg University, Heidelberg, Germany; 8 Recognized Research Group in Optical Diagnostic Techniques, University of Valladolid, Valladolid, Spain; 9 Department of Ophthalmology and Sciences of Vision, University of Oviedo, Oviedo, Spain; Wenzhou Medical University Eye Hospital, CHINA

## Abstract

**Purpose:**

To highlight the potential shortcomings associated with the current use Rasch analysis for validation of ophthalmic questionnaires, and to present an alternative application of Rasch analysis to derive insights specific to the cohort of patients under investigation.

**Methods:**

An alternative application of Rasch analysis was used to investigate the quality of vision (QoV) for a cohort of 481 patients. Patients received multifocal intraocular lenses and completed a QoV questionnaire one and twelve months post-operatively. The rating scale variant of the polytomous Rasch model was utilized. The parameters of the model were estimated using the joint maximum likelihood estimation. Analysis was performed on data at both post-operative assessments, and the outcomes were compared.

**Results:**

The distribution of the location of symptoms altered between assessments with the most annoyed patients completely differing. One month post-operatively, the most prevalent symptom was starbursts compared to glare at twelve months. The visual discomfort from the most annoyed patients is substantially higher at twelve months. The current most advocated approach for validating questionnaires using Rasch analysis found that the questionnaire was “Rasch-valid” one month post-operatively and “Rasch-invalid” twelve months post-operatively.

**Conclusion:**

The proposed alternative application of Rasch analysis to questionnaires can be used as an effective decision support tool at population and individual level. At population level, this new approach enables one to investigate the prevalence of symptoms across different cohorts of patients. At individual level, the new approach enables one to identify patients with poor QoV over time. This study highlights some of the potential shortcomings associated with the current use of Rasch analysis to validate questionnaires.

## 1 Introduction

The concept of patient-reported outcome (PRO) measures borrowed from clinical trials have become nowadays a routine practice in ophthalmology. Patients are invited pre and post-operatively to complete PRO instruments, most commonly questionnaires, whose data are used to gain more insight into the patient’s own experience of vision-specific health-related problems as well as the impact of ophthalmic treatments on their quality of life. The overall aim of this exercise is to improve the clinical quality of care.

During the last decade, Rasch analysis [1]–[3] has been used not only to assess and define the subscale structure of items within ophthalmic questionnaires [4]–[9], but also to systematically dismiss the relevance of certain questionnaires solely on statistical grounds rather than substantive grounds [10]–[18]. However, the major shortcoming of such applications of Rasch analysis is the oversight on some fundamental assumptions enabling the key commendable features of the Rasch model as well as the intrinsic nature of the phenomena a questionnaire is attempting to measure.

Some of the most fundamental hypotheses, assumed by the Rasch model, include the homogeneity assumptions for both the test items (the questionnaire in this case) and the population of interest (the patients in this case). These two assumptions have been instrumental in the derivation of the Rasch model. Indeed, they enable the decomposition of the probability of item responses into two independent components, namely an item-specific difficulty parameter, which is constant across all the population of interest, and an ability parameter for each individual, which is identical across all the items in the test. This principle of invariant comparison was termed “specific objectivity” by Rasch [2], [19].

The assumptions of homogeneity, i.e. an underlying unidimensional structure among the patients, is less likely to be met in sections of the data collected via ophthalmic questionnaires for various reasons. For instance, some or all of the items of the questionnaire may function differently in patient subpopulations, or the responses of patients to these items may depend on more than one underlying construct or latent trait. This could be problematic in particular when the questionnaires are completed by a population of patients from different backgrounds, for instance in terms of lifestyle or by the same population of patients at different time points. The aforementioned eventualities may readily deviate the response to the questionnaires from the assumption of “specific objectivity” [2], [19], which is crucial for a proper application of the Rasch model. As a consequence, a so-called “Rasch validated” questionnaire for a given cohort of patients and a given latent trait may not be “Rasch-valid” for another cohort of patients with the same latent trait, or for the same cohort of patients with the same latent trait at a different time point. The approach currently advocated for validating ophthalmic questionnaires is entirely based on the analysis of fit of the Rasch model on data from a single potentially non representative cohort of patients, occasionally with a relatively small sample size e.g. [10]–[12], [16], [20], [21]. However, it is well recognized that the analysis of fit for the Rasch model is a never ending process since a continued use of the instrument requires constant monitoring of the item and person responses to maintain quality control [22].

At its inception, the Rasch model aims to assess psychometric properties of some intelligence and attainment tests. In such context, individuals are examined via some tests consisting of several items. When there is sufficient similarity among the individual in the way they approach the tests, then the responses to the items are expected to follow some specific patterns. The individuals misfitting the model correspond to those individuals whose responses deviated from the expected patterns and it could be envisaged that these responses are partially based on guessing or they are due to some carelessness from the respondents. On the other hand, items misfitting the model can be interpreted as follows: either the items do not contribute to an adequate assessment of the examinees or there is an underlying multi-dimensional structure among the individuals. However, the misfit statistics on their own do not provide enough ground to remove items from the tests. On the contrary, misfitting items are worth keeping since they provide useful information on the underlying dimensional structure among the individuals.

In the context of test-based ophthalmic instruments such as LogMAR or Snellen charts for visual acuity testing, the responses to the items are sufficiently similar among patients with similar visual function. Hence, the responses are expected to follow some specific patterns, and serious item misfit generally indicates an unanticipated problem which may be attributed to the quality of the items. However, for ophthalmic questionnaires which are based on items often independent, the misfitted items may be due to various reasons including an underlying multi-dimensional structure among the patients. For instance, a consistent difference in response propensity introduced by variation in the characteristics of the respondents such as lifestyle, age and gender may contribute significantly to item and/or person misfits. The misfitted items and/or persons therefore may not necessarily be outliers. Even if they were, medical care implies that patients are taken as individuals with their own problems, and not as a group. Furthermore, misfitting items may actually be relevant for the quality of care (although they may imply a different latent trait). In other words, Rasch validation as performed currently, might help qualify a technique or a therapy but it does not provide any insight into the cause of particular patients being affected differently by the same item.

Issues associated with the removal of items are well known in the Rasch analysis literature see e.g. [23, 24] and the references therein. For instance, it is well known that removing items from a questionnaire is very likely to increase the intrinsic variance within the data, which could affect the estimation of the person and item measures. This could be problematic, in particular when comparing items/persons across different conditions. Moreover, removing some items could misfit other items which were not initially misfitted, leading to a downward spiral in the number of items in the questionnaire. Massof et al. [25] presented a study on a visual function questionnaire in which they maintained misfitted items and provided a meaningful interpretation of the items. They showed that items with infit statistics greater than 2.5 the standard deviation from the expected value are related to mobility tasks, whereas items with infit statistics lower than 2.5 the standard deviation from the expected value are associated with reading tasks. Furthermore, leveraging on these misfit statistics, the authors used Principal Component Analysis to demonstrate the non-unidimensional aspect of the visual function trait under investigation.

In contrast with the current validation practice, which consists of using Rasch analysis to dismiss [9]–[18] or approve [9], [14], [21]–[30] an ophthalmic questionnaire based solely on the misfit statistics of the items, this work introduces an alternative, meaningful and relevant application of the Rasch model to analyze data collected via ophthalmic questionnaires. The proposed approach aims to present Rasch analysis as a decision support tool for deriving valuable insights specific to the cohort of patients under investigation, at both population and individual level. At the population level, such an approach enables the investigation of the prevalence of ophthalmic symptoms across different cohorts of patients pre- operatively and post-operatively, in order to assess the effectiveness of a treatment—e.g. different types of intraocular lenses (IOLs) or different surgical procedures. At the individual level, the new approach can be applied across a population at different time points and identify patients who experienced most visual discomfort pre- operatively and/or post-operatively, so that additional appropriate care and monitoring can be dedicated to them. Ultimately, this new perspective will pave the way for a more adequate application of Rasch analysis within the context of ophthalmic questionnaires, so that insights gained from the analysis can be exploited to enhance the quality of care and patient care experience. However, this paper does not attempt to advocate an alternative method of validation of ophthalmic questionnaires, and our future work will investigate this aspect of ophthalmic questionnaire development.

The remaining part of this paper is organized as follows. Section 2 briefly presents the Rasch model and highlights the key mathematical features and their meaning. Then, a brief overview and illustration of Rasch analysis for dichotomous response data is provided. Section 3 presents an application of Rasch analysis on data from an ophthalmic questionnaire as an effective decision support tool for a post-operative follow-up of patients, at both population and individual level. The overarching aim of the process is to improve our understanding of how patients’ responses to the questionnaire evolve over time, which ultimately should provide the opportunity to improve the patient care experience. Finally, Section 4 concludes the paper and highlights some potential further research.

## 2 Background

In a series of seminal research works [1]–[19] Rasch introduced a probabilistic framework for analyzing the ability of pupils using a model for the items of a test, which is known as the Rasch Model. This section will briefly present a basic set of assumptions and the general framework that underpins the Rasch model from its original form to its most commonly used version, implemented in most of the software packages dedicated to Rasch Analysis.

**Fig 1:**
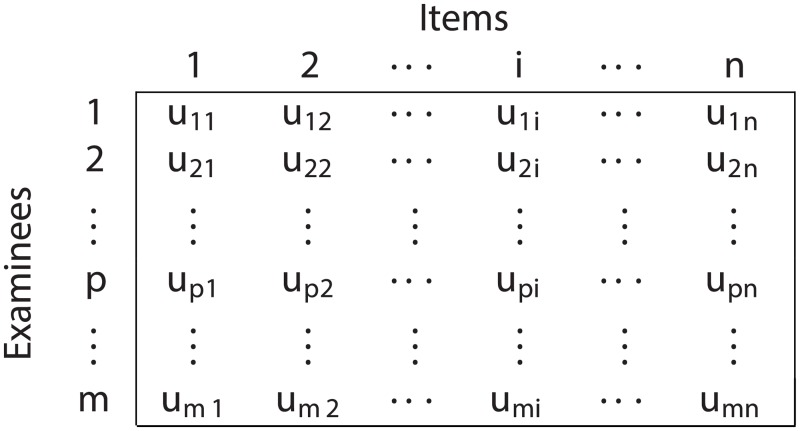
General form of the data matrix for the Rasch model.

The Rasch model formulation is based on a two-dimensional data matrix, denoted *U*, obtained by administering a test, which consists of *n* items, to *m* examinees or persons. Each component *u*_*pi*_, of the matrix *U*, denotes the response of the examinee or the person *p* to the item *i*. The response to the items, i.e. *u*_*pi*_, can be dichotomously or polytomously scored hence the denomination *dichotomous* or *polytomous* Rasch model, respectively. The general form of the data matrix *U* is shown in Fig 1. The Rasch model [1], [3] owes its key desirable mathematical features to a certain number of assumptions, and the most fundamental assumptions will be described in this section.

The fundamental assumptions behind the Rasch model are:

**Assumption 1** [1, 31] *The response of an examinee or a person p to an item i, u*_*pi*_, *depends solely on the examinee’s ability, characterized by the parameter a*_*p*_, *and the difficulty of the item, characterized by the parameter d*_*i*_.

**Fig 2:**
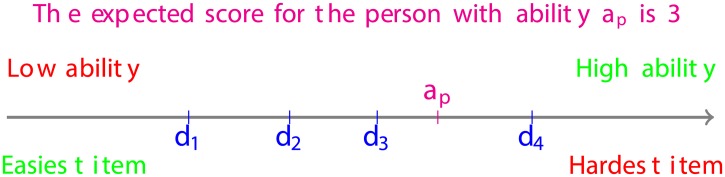
Examinees-items map along the line characterizing the underlying continuum latent trait.

Basically, the main purpose of a test is to estimate the location of an individual with a certain ability, taking the test, on the line defined by the difficulty level of the different test items [31]. This is illustrated in Fig 2, where the ability of the person *p* is between *d*_3_ and *d*_4_, which represent the difficulty level of items 3 and 4, respectively. Therefore, it is expected that the person *p* will be able to answer correctly all the items with difficulty below his/her ability *a*_*p*_. If the score for a correct answer to each item is 1, then the total expected score for the person *p*, from this test, is 3.

**Assumption 2** [1, 3] *The ability and the difficulty characterize the person and the item, respectively, such that if an examinee p was k times as able as an examinee q then a*_*p*_ = *ka*_*q*_. *Similarly, if an item i was k times as difficult as an item j, then d*_*i*_ = *kd*_*j*_. *Thus*,

$$\begin{array}{c} \frac{a_{p}}{d_{i}}=\frac{a_{q}}{d_{j}}. \end{array}$$ (1)

Using Eq (1) in Assumption 2, Assumption 1 reduced to the following.

**Assumption 3 (Unidimensionality)** *The response of an examinee p to an item i, u*_*pi*_, *depends solely on the ratio* $\frac{a_{p}}{d_{i}}$, *denoted ξ*_*pi*_.

Another key assumption behind the Rasch model is that:

**Assumption 4 (Specific objectivity)** [3] *For any given set of items with some given difficulties and any population of examinees with some given abilities, the response of the examinees to the items are stochastically independent*.

This assumption considers that on the one hand, the response of some examinees with the same ability to the *n* items in the test are independent. On the other hand, the response of the examinees to an item with a given difficulty are independent. Thus, this assumption enables the Rasch model to treat the examinees and the items independently. However, this assumption is not always satisfied in practice.

### 2.1 Dichotomous Rasch model

If the responses to test items consist of only two categories then without loss of generality we can assume that the response of any examinee *p* to any item *i*, *u*_*pi*_, can only be either 0 or 1. The dichotomous Rasch model estimates the probability of any instance of response *u*_*pi*_ as:

$$\begin{array}{c} P\left(u_{pi}|{\hat{a}}_{p},{\hat{d}}_{i}\right)=\frac{e^{\left({\hat{a}}_{p}-{\hat{d}}_{i}\right)u_{pi}}}{1+e^{\left({\hat{a}}_{p}-{\hat{d}}_{i}\right)}}, \end{array}$$ (2)

where ${\hat{a}}_{p}$ is the estimated ability of the person *p* and ${\hat{d}}_{i}$ is the estimated difficulty of item *i*.

Some details on the derivation of the dichotomous Rasch model as well as its mathematical properties are presented in Appendix A.

#### Parameters estimation and goodness of fit measures for the Rasch model

**Estimating parameters of the Rasch model**. There are a variety of methods which can be used to estimate the set of parameters (${\hat{a}}_{p},{\hat{d}}_{i}$) of the Rasch model (2), see [32], [33] for an overview. However, the most commonly implemented methods in software packages dedicated to Rasch analysis include the joint maximum likelihood estimation (JMLE) and the marginal maximum likelihood estimation (MMLE).

The JMLE procedure assumes some initial known estimates of the parameters of the persons and items, then uses Newton-Raphson iterations to improve jointly the estimates of parameters, until a specific convergence criterion has been satisfied. This approach requires the removal of items and persons with perfect scores (i.e. all their scores are either equal to one or equal to zero for the dichotomous model).

The MMLE approach assumes a known distribution, of the persons’ parameters, which is used to estimate the items’ parameters. In contrast with the JMLE approach, MMLE enables estimation of the parameters of items and persons with all scores equal to one or zero. However, the reliability of the parameters estimated using the MMLE approach depend upon the relevance of the assumed distribution of the person parameters. Hence, the MMLE approach could be prone to greater bias compared to the JMLE approach.

**Measuring goodness of fit for the Rasch model**. The most commonly used goodness of fit measure for the Rasch model, i.e. how well the observed data fit the model, is to test the normality of residuals. Each residual represents a piece of information not covered by the model, and large residuals raise doubts about the match between the model and data [31], [34].

In Rasch analysis, the goodness of fit measures, also called misfits statistics, consist of the infit and outfit test statistics which are based on the standardized residuals. The outfit statistic, also referred to as outlier-sensitive fit statistic, is a measure that is sensitive to unexpected observations by persons on items that are very easy or very hard for them, and vice-versa. The infit statistic, also referred to as inliner-pattern-sensitive fit statistic, is a measure that is sensitive to unexpected patterns of observations by persons on items that are targeted for them, and vice-versa. The most commonly used misfit statistics for Rasch analysis are the Mean-squares misfit statistics and *z*-standardized misfit statistics. Some details on the derivation of these statistics are provided in Appendix B.

Mean-squares fit statistics (Outfit MNSQ and Intfit MNSQ) describe the level of the randomness in the response data, and their expected values are 1. The values of mean-squares fit statistics which are very low compared to 1, indicate a high degree of predictability of responses to the items by the model, i.e. the model overfits the data. On the other hand, the values of mean-squares fit statistics, which are very high compared to 1, indicate a high degree of unpredictability of responses to the items by the model, i.e. the model provides a distorted representation of the data. A general guideline is that values of mean-squares fit statistics greater than 1.5 suggest a deviation of the model from the unidimensionality assumption within the data. The value 1.5 is rather a rough approximation of the z-score for an area of 0.95 (or 95%) for the cumulative function of the standard normal distribution, which is about 1.64. This means that 95% of the values of mean-squares fit statistics are generally below the threshold of 1.5 (or to be more accurate 1.64). On the other hand, values of mean-squares fit statistics less than 0.5 suggest an overfitting of the model.

The *z*-standardized misfit statistics describe the improbability of the model to fit the data, and their expected values are 0. The values of *z*-standardized misfit statistics which are very low compared to 0 (less than -1.9) indicate an overfitting of the model; on the other hand, the values of *z*-standardized misfit statistics which are very high compared to 0 (greater than 1.9) indicate that the model is less likely to fit the data. The *z*-standardized misfit statistics are generally used when the mean-squares statistics fail.

### 2.2 Polytomous Rasch model

When the item response data have more than two response options, a generalized version of the Rasch model known as the polytomous Rasch model is used. The polytomous Rasch model inherits most of the properties of the dichotomous Rasch model. The main difference between these two models lies in the introduction of the concept of thresholds in the polytomous version. These thresholds play an important role for a polytomous model since they enable the identification of critical points along the latent trait continuum. Furthermore, for a polytomous model, each item response category has a unique probability distribution associated with it, and at a threshold the relative probabilities of two adjacent item response categories are equal.

There are two types of polytomous Rasch models commonly used in the literature. Namely, the Rating Scale Model (RSM) [35], in which the threshold estimate, for a given category response, is identical for all the items, and the Partial Credit Model (PCM) [36], in which the estimates of the thresholds can vary across the items and response categories. The PCM model can be viewed as a generalization of the RSM.

The RSM can be formulated as follows:

$$\begin{array}{c} P\left(u_{pi}=\eta |{\hat{a}}_{p},{\hat{d}}_{i},{\hat{h}}_{t}\right)=\frac{e^{\sum_{t=0}^{\eta }\left({\hat{a}}_{p}-{\hat{d}}_{i}+{\hat{h}}_{t}\right)}}{\sum_{\eta =0}^{k}e^{\sum_{t=0}^{\eta }\left({\hat{a}}_{p}-{\hat{d}}_{i}+{\hat{h}}_{t}\right)}}, \end{array}$$ (3)

where, *p* = 1, …, *m* denotes an examinee’s index, *i* = 1, …, *n* denotes an item’s index, *η* = 0, …, *k* denotes an item response category, and *t* = 0, …, *k* − 1 denotes a threshold’s index; the parameter ${\hat{h}}_{t}$ denotes the common threshold associated with all the items for the category response *t*, whereas the rest of the parameters are identical to those defined for the dichotomous model in the previous sections.

The PCM can be formulated as follows:

$$\begin{array}{c} P\left(u_{pi}=\eta |{\hat{a}}_{p},{\hat{d}}_{it}\right)=\frac{e^{\sum_{t=0}^{\eta }\left({\hat{a}}_{p}-{\hat{d}}_{it}\right)}}{\sum_{\eta =0}^{k}e^{\sum_{t=0}^{\eta }\left({\hat{a}}_{p}-{\hat{d}}_{it}\right)}}, \end{array}$$ (4)

where ${\hat{d}}_{it}$ denotes the joint item difficulty and threshold parameter, while the remaining notations are identical to those in the RSM model (3).

### 2.3 A brief overview and illustration of Rasch analysis for dichotomous response data

From the above sections, Rasch analysis can be summarized into the following three main steps:

Step 1 This step uses the data from the response matrix to estimate the initial values of the difficulty and ability parameters for each item and person, respectively;
Step 2 This step uses the initial parameters estimates, from Step 1, to obtain some optimal estimates of the difficulty of items and the ability of persons parameters; the most commonly used techniques to achieve this include the joint maximum likelihood estimation (JMLE) and the marginal maximum likelihood estimation (MMLE);
Step 3 This step consists of the identification of items and persons with unexpected response patterns using goodness of fit measure, e.g. Mean Square or *z*-standardized misfit statistics.

Steps 1 and 2 are often combined into a single step known as the *calibration* step, whereas the last step is generally termed the *fit analysis*.

#### Illustration of Rasch analysis to assess a LogMAR chart for visual acuity testing

**Fig 3:**
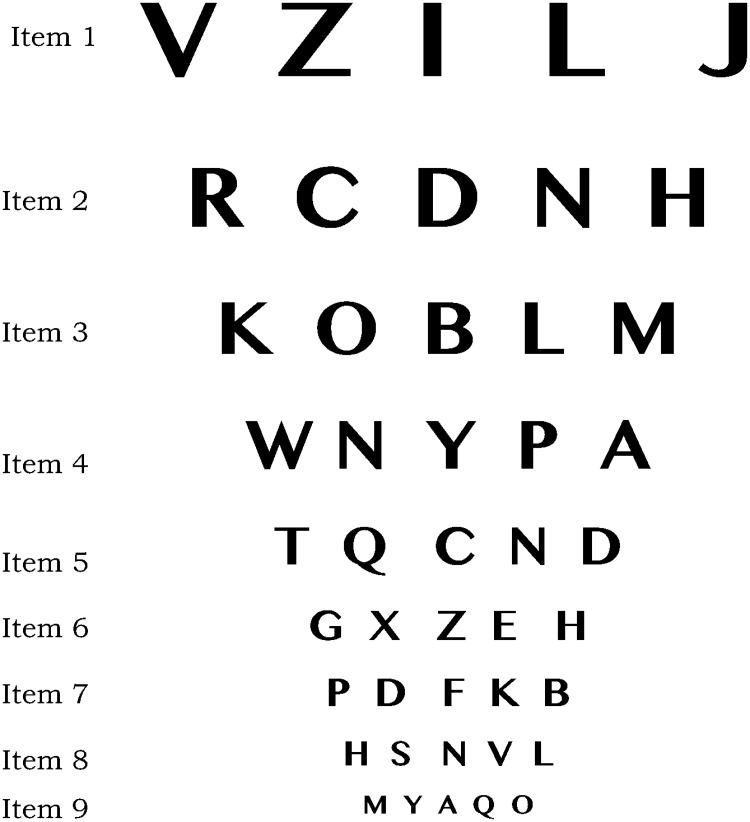
A LogMAR chart for visual acuity testing, with 9 items.

In order to illustrate the aforementioned steps, we consider the following data matrix for dichotomous response where 10 patients undergo a visual acuity test using the 9 items LogMAR chart depicted in Fig 3. In the response data matrix, the score of 1 corresponds to a correct answer to an item (i.e. if at least 3 correct answers are given in a row of the chart) by a patient, whereas a score of 0 corresponds to an incorrect answer (i.e. if at most 2 correct answers are given in a row of the chart). In this situation, the concept of person ability and item difficulty, in the Rasch model, corresponds to the patient’s location (in logit) in terms of *visual acuity*, and the item’s location (in logit) in terms of *difficulty to read*. The higher the location of a patient (respectively, an item), the higher the visual acuity (respectively difficulty to read) of the patient (respectively for the item).

**Remark 1** *Due to the following conditions, Rasch analysis can be an appropriate approach for the assessment of a LogMAR chart for visual acuity testing*:

1. *for a LogMAR chart, the responses to items are sufficiently similar between patients with similar visual function*;
2. *the responses to the items are expected to follow specific patterns according to the patient’s location, in terms of visual acuity, and the item’s location, in terms of difficulty to read; for instance, a patient with a given location is expected to read correctly most of the items with lower locations, and the misfit statistics (e.g. Outfits and Infits MSNQ) enable the identification of any unexpected response patterns from a patient and for an item*;
3. *the scenario complies with the most fundamental assumptions behind the Rasch model (namely, Assumptions 1, 2, 3, and 4)*.

#### Step 1: Estimation of the initial locations for items and patients

In this step, the following rows and columns in Table 1 are calculated: columns $r_{p},\mu_{p},{\hat{a}}_{p}^{0}$ and rows $r_{i},\mu_{i},{\hat{d}}_{i}^{0},{\hat{d}}_{i}^{0(Adj)}$, where

- $r_{p}=\sum_{i=1}^{n}u_{pi}$ and $r_{i}=\sum_{p=1}^{m}u_{pi}$ are the total score for patient *p* and item *i*, respectively;
- $\mu_{p}=\frac{r_{p}}{n}$ and $\mu_{i}=\frac{r_{i}}{m}$ are the proportions of the correct responses for patient *p* and item *i*, respectively;
- ${\hat{a}}_{p}^{0}=\text{log}(\frac{\mu_{p}}{1-\mu_{p}})$ and ${\hat{d}}_{i}^{0}=\text{log}(\frac{1-\mu_{i}}{\mu_{i}})$ are the initial estimates of the locations for patient *p* and item *i*, respectively;
- ${\hat{d}}_{i}^{0(Adj)}={\hat{d}}_{i}^{0}-\frac{\sum_{i=1}^{n}{\hat{d}}_{i}^{0}}{n}$, is the adjusted initial location for item *i*; thus the mean of the adjusted locations for the items is zero.

**Table 1:** The dichotomous response data matrix from a visual acuity test using the LogMAR chart in Fig 3, and the corresponding location estimates for patients and items.

Items	Item 1	Item 2	Item 3	Item 4	Item 5	Item 6	Item 7	Item 8	Item 9	*r*_*p*_	*μ*_*p*_	${\hat{a}}_{p}^{0}$	${\hat{a}}_{p}^{*}$
Patient													
Patient 1	0	1	1	1	1	1	1	1	0	7	0.78	1.25	1.84
Patient 2	1	1	1	1	1	1	1	0	1	8	0.89	2.08	2.98
Patient 3	1	1	1	1	1	1	0	1	0	7	0.78	1.25	1.84
Patient 4	1	1	1	1	1	1	0	1	0	7	0.78	1.25	1.84
Patient 5	1	1	1	1	1	1	0	1	0	7	0.78	1.25	1.84
Patient 6	1	1	1	1	1	0	1	0	0	6	0.67	0.69	0.94
Patient 7	1	1	1	1	0	1	0	0	0	5	0.56	0.22	0.18
Patient 8	1	0	1	0	1	0	0	0	0	3	0.33	-0.69	-1.12
Patient 9	0	1	0	1	0	0	0	0	0	2	0.22	-1.25	-1.81
Patient 10	0	0	0	0	0	1	0	0	1	2	0.22	-1.25	-1.81
*r*_*i*_	7	8	8	8	7	7	3	4	2				
*μ*_*i*_	0.7	0.8	0.8	0.8	0.7	0.7	0.3	0.4	0.2				
${\hat{d}}_{i}^{0}$	-0.85	-1.39	-1.39	-1.39	-0.85	-0.85	0.85	0.41	1.39				
${\hat{d}}_{i}^{\text{0(Adj)}}$	-0.39	-0.93	-0.93	-0.93	-0.39	-0.39	1.30	0.86	1.83				
${\hat{d}}_{i}^{*}$	-0.61	-1.45	-1.45	-1.45	-0.61	-0.61	2.03	1.42	2.72				

#### Step 2: Estimation of the optimal locations for items and patients

In this step, the initial estimates of the visual acuity location of each patient, ${\hat{a}}_{p}^{0}$, and initial adjusted estimates of the difficulty location of each item, ${\hat{d}}_{i}^{0(Adj)}$, are improved by maximizing the likelihood of the response of each patient to each item to obtain the optimal patient’s visual acuity location (${\hat{a}}_{p}^{*}$) and item difficulty location (${\hat{d}}_{i}^{*}$). The joint maximum likelihood estimation (JMLE) was used to obtain the optimal parameters ${\hat{a}}_{p}^{*}$ and ${\hat{d}}_{i}^{*}$.

#### Step 3: Identification of items and patients with unexpected response patterns

**Fig 4:**
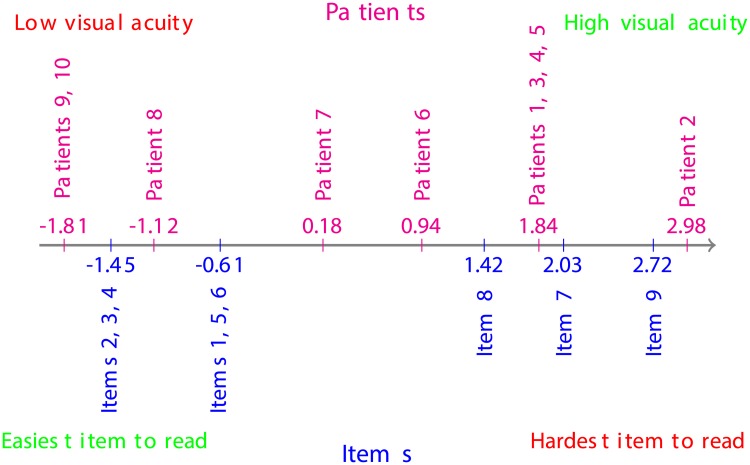
Patient-item map along the line characterizing their locations (in logit), in terms of visual acuity and difficulty to read, respectively.

From the optimal locations results for items and patients, presented in Tables 2 and 3 and depicted in Fig 4, the following observations can be drawn for this cohort of patients.

- The most difficult item to read was Item 9, followed by Item 7 and Item 8, respectively, whereas Items 2, 3, 4 were the easiest to read, followed by Items 1, 5, 6. Furthermore, since Items 2, 3, 4 have the same values for the location estimates, then the model suggested that these three items have the same degree of difficulty for this cohort of patients. Likewise, Items 1, 5, 6 have the same degree of difficulty for this cohort of patients.
- The patient with the highest visual acuity within the cohort was Patient 2, followed by Patient 1, 3, 4, 5, Patient 6 and Patient 7, respectively, whereas Patient 9, 10 had the lowest visual acuity, followed by Patient 8. Although Patient 2 did not answer Item 8 correctly, he/she is most likely to respond correctly to all the items, thus his/her location is higher than the location of the hardest item (Item 9). On the other hand, although Patients 9 and 10 responded correctly to two items, the erratic patterns in their responses suggest that they are less likely to answer correctly any of the items on the chart. Thus, the estimates of their locations are lower than the location of the easiest item to read.
- The relatively high Outfit MNSQ value, compared to 1, for Item 9 reflected the outlying response pattern for this item. In fact, only the patient with the highest visual acuity (Patient 2) and one of the patients with the lowest visual acuity (namely Patient 10) responded correctly to this item. This is a rather unexpected response pattern for Item 9.
- The relatively high Outfit and Infit MNSQ values, compared to 1, for Patient 10, highlighted the outlying patterns of his/her responses. Indeed, this patient answered correctly only one relatively easy item (Item 6) and the hardest item (Item 9), which is unexpected.
- The relatively high Outfit MNSQ value, compared to 1, for Patient 1, indicated that this patient only failed the hardest item (Item 9) and a relatively easy item (Item 1). The latter wrong response is rather unexpected.

**Table 2:** Estimates of item location (in logits), in terms of difficulty to read, and the corresponding standard error, mean square (MNSQ) infits and outfits. The fit statistics, highlighted in bold, are those exceeding the threshold of 1.5.

Item ID	Item location (in logit)	Standard Error	Outfits MNSQ	Infits MNSQ
Item 1	-0.61	0.89	1.47	1.18
Item 2	-1.45	0.96	0.4	0.82
Item 3	-1.45	0.96	0.26	0.51
Item 4	-1.45	0.96	0.4	0.82
Item 5	-0.61	0.89	0.51	0.81
Item 6	-0.61	0.89	0.97	1.25
Item 7	2.03	0.80	0.73	1.00
Item 8	1.42	0.78	0.85	0.92
Item 9	2.72	0.87	**9.46**	1.18

**Table 3:** Estimates of patient location (in logit), in terms of visual acuity, and the corresponding standard error, mean square (MNSQ) infits and outfits. The fit statistics, highlighted in bold, are those exceeding the threshold of 1.5.

Patient ID	Patient location (in logit)	Standard Error	Outfits MNSQ	Infits MNSQ
Patient 1	1.84	0.99	**1.56**	1.38
Patient 2	2.98	1.19	0.67	1.36
Patient 3	1.84	0.99	0.25	0.46
Patient 4	1.84	0.99	0.25	0.46
Patient 5	1.84	0.99	0.25	0.46
Patient 6	0.94	0.91	1.02	1.23
Patient 7	0.18	0.84	0.47	0.58
Patient 8	-1.12	0.80	0.84	1.14
Patient 9	-1.81	0.87	0.50	0.77
Patient 10	-1.81	0.87	**10.89**	**1.65**

## 3 Application of Rasch analysis to assess data from an ophthalmic PRO instrument

Test-based ophthalmic instruments, such as visual acuity tests using Snellen or LogMAR charts, where the responses to the items are sufficiently similar among patients, and expected to follow specific patterns,—comply with the main assumptions behind the Rasch model. Therefore, the model can be used to assess whether these instruments are appropriate for their purpose. In such cases, serious item misfit generally indicates an unanticipated problem which may be attributed to the quality of the items.

However, in the context of ophthalmic questionnaires, the unidimensionality assumption of the responses to the items is not always satisfied and as a consequence, some of the major assumptions of the Rasch model, namely Assumptions 3 and 4 presented in section 2 do not always fully hold. Due to the nature of the responses, which encompass any potential underlying multi-dimensional structure among the patients, the misfits statistics may be interpreted differently. For instance, a consistent difference in response propensity introduced by various respondents’ characteristics such as lifestyle, age and gender may contribute significantly to items and/or person misfits.

The currently most advocated practice, for validating ophthalmic PRO questionnaires, is either to collapse some item response categories or to drop items or questions which misfit the Rasch model [4], [10], [26]. If for any reason all the items misfit the model or some estimation problems are encountered during the process then the entire questionnaire is dismissed [9]–[18]. However, even for tests based on items where responses are sufficiently similar between patients, it is well recognized that in order to maintain quality control, a continuous monitoring of items and patient responses is required [22].

The main objective of this study is to attempt to introduce an alternative application of Rasch analysis, which is specific to the cohort under investigation, as an alternative to the current misuse of the method to dismiss [9]–[18] or approve [9], [14], [21]–[30] a questionnaire based on the misfit statistics of data from a single and potentially non-representative cohort of patients, occasionally with a relatively small sample size e.g. [10]–[12], [16], [20], [21].

In this section, we will present a case study to illustrate how the proposed approach enables the use of Rasch analysis as a decision support tool for post-operative patient follow-up, in order to improve patient care experience.

### 3.1 The PRO instrument

The PRO instrument, used for this study, is a previously developed Quality of Vision (QoV) questionnaire [7], from which only the bothersome scale was used to reduce number of questions. The questionnaire attained information on the presence of various dysphotopsias and visual disturbances that a patient may experience, and the annoyance of each side effect to the patient. Patients reported the degree of annoyance of the nine vision related symptoms presented in Table 4. The choice of these nine symptoms was motivated by their substantive representativeness of QoV. This QoV questionnaire uses pictures to further aid understanding of the dysphotopsias or visual disturbances being questioned. A sample of the pictures used is provided in Supporting Information (S1 Fig). In addition to the original questionnaire a linear 0-10 scale was incorporated to define each patients own view of their overall QoV, in order to gain a better understanding of post-operative satisfaction.

**Table 4:** QoV questionnaire: Symptoms, questions and response options.

Symptom label	Symptom denomination	Questions	Response Options
GL	Glare	How bothersome is the glare?	Not at all, A little, Quite, Very
HL	Haloes	How bothersome are the haloes?	Not at all, A little, Quite, Very
ST	Starburst	How bothersome are the starbursts?	Not at all, A little, Quite, Very
HV	Hazy vision	How bothersome is the hazy vision?	Not at all, A little, Quite, Very
BV	Blurred vision	How bothersome is the blurred vision?	Not at all, A little, Quite, Very
DS	Distortion	How bothersome is the distortion?	Not at all, A little, Quite, Very
DI	Double image	How bothersome are the double images?	Not at all, A little, Quite, Very
FL	Fluctuation	How bothersome is the fluctuation in your vision?	Not at all, A little, Quite, Very
DDP	Difficulty in depth perception	How bothersome is the difficulty in depth perception?	Not at all, A little, Quite, Very

**Remark 2** *In contrast with conditions in Remark 1 for the assessment of the LogMAR chart for visual acuity test, for ophthalmic PRO questionnaires in general and the QoV questionnaire in particular, the context is as follows*.

1. *the responses, to the questions associated to the symptoms listed in the QoV questionnaire, reflected the feeling of the patient regarding these symptoms*;
2. *the symptoms, listed in the QoV questionnaire, are not ordered at all, since they are assumed to be totally independent; therefore, there is no specific response pattern expected according to patients and symptoms’ locations; for instance, a patient with a given location value is not expected to be affected by a symptom with a lower location; therefore the outfit and infit statistics need to be interpreted differently in this context, as described in section 3.3*;
3. *the scenario does not fully comply with some of the key assumptions behind the Rasch model, in particular Assumptions 3 and 4; therefore, the latent trait of interest may not be unidimensional*.

### 3.2 Participants

The participants consist of a cohort of 481 patients who had implantation of multifocal intraocular lenses (IOLs) from Cathedral Eye Clinic, Belfast. Patients were thoroughly assessed and informed of the risks of the procedure and all patients gave their informed consent for their anonymized data to be used for research purposes.

The patients received multifocal IOLs following either refractive lens exchange (RLE) or cataract extraction surgery. Full ophthalmologic examination was performed on each patient approximately one month and one year post-operatively following the implantation of the IOLs. In each case the QoV questionnaire was completed with an optometrist to ensure understanding of the questions.

The summary statistics of the patients are presented in Table 5. Among these 481 patients, 125 and 160 declared not suffering at all from any of the nine symptoms, one month and one year post-operatively, respectively. Therefore, these patients have been discarded from the analysis so that the JMLE method operates properly.

**Table 5:** Participants’ demographics.

Characteristics	Result
Mean age (in years) ± SD	62 ±9.00
Age range (in years)	30-93
Sex, Number (%)	
Male	195 (40.5%)
Female	286 (59.5%)

### 3.3 Contextualization of the Rasch model

In order to properly interpret the outputs of the Rasch model we need to establish the meaning of the terminologies used in Rasch analysis within the context of the ophthalmic questionnaire of interest. In this context,

1. the ability parameter ${\hat{a}}_{p}$, associated to an examinee *p* in the Rasch model, corresponds to the location (in logit), in terms of *perception of visual discomfort*, for the patient *p*; the lower the value of this parameter the lower the perception of visual discomfort, whereas the higher the value of the parameter the higher the perception of visual discomfort.
2. the difficulty parameter ${\hat{d}}_{i}$, associated to an item *i* in the Rasch model, corresponds to the location (in logit), in terms of *“non-prevalence” within the cohort*, for the symptom *i*; the lower the value of this parameter the higher the proportion of patients within the cohort affected by the symptom, whereas the higher the value of the parameter the lower the proportion of patients affected by the symptom.
3. the probability for a patient *p* to give a response category *η* to the question associated with symptoms *i*, given her/his location, in terms of his/her perception of visual discomfort, ${\hat{a}}_{p}$, and the location of the symptom, in terms of its prevalence within the cohort, ${\hat{d}}_{i}$, is as follows:
  $$\begin{array}{c} P\left(u_{pi}=\eta |{\hat{a}}_{p},{\hat{d}}_{i},{\hat{h}}_{t}\right)=\frac{e^{\sum_{t=0}^{\eta }\left({\hat{a}}_{p}-{\hat{d}}_{i}+{\hat{h}}_{t}\right)}}{\sum_{\eta =0}^{k}e^{\sum_{t=0}^{\eta }\left({\hat{a}}_{p}-{\hat{d}}_{i}+{\hat{h}}_{t}\right)}}, \end{array}$$ (5)
  where the parameter ${\hat{h}}_{t}$ denotes the common threshold associated with all the items for the category response *t*.

The calibration step of Rasch analysis enables the researcher at a glance to compare and contrast different populations and define whether the examined items hold the same weight or relevance within the particular cohort. An example might be how car drivers are affected by glare compared to non car drivers. Different positioning of the items might at a glance highlight the differential importance of glare in these two different populations of patients. The fit analysis however would help to quickly highlight individuals potentially with ocular problems including higher levels of astigmatism or macular problems such as cystoid macular œdema (CMO) or age-related macular degeneration (AMD), which might produce values of the misfit statistics deviating significantly from the expected values for the Rasch model.

### 3.4 Results and discussion

The types of different response categories for the questions, described in Table 4, suggest a polytomous Rasch model as the most appropriate option. The Rating Scale Model (RSM) [35] was used to analyse the questionnaire data, and the parameters of the model were estimated by mean of the joint maximum likelihood estimation (JMLE) method, implemented using the Matlab^®^ software [37].

The objective of the analysis was not to select only symptoms which fit the Rasch model but to ensure that most of the symptoms affecting the QoV, in general, are covered as suggested by Messick [23]. Furthermore, the interpretation of the outputs of the model is specific to the data of the response matrix under investigation.

#### Analysis of the questionnaire data collected one month post-operatively

**Fig 5:**
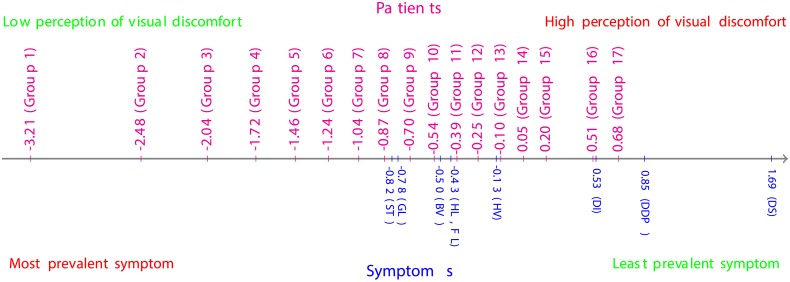
Patient-symptom map for questionnaire data collected one month post-operatively.

**Fig 6:**
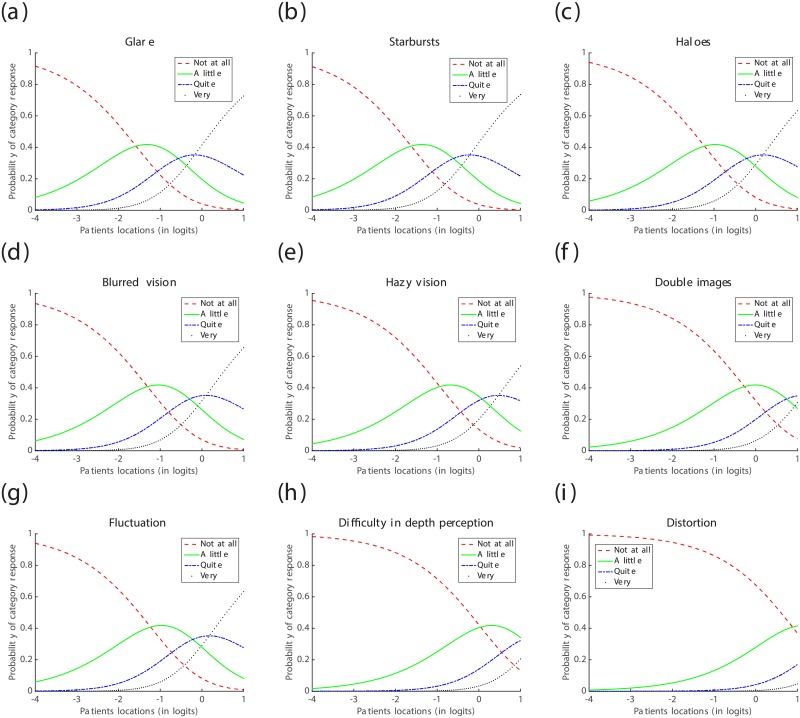
Item Characteristics Curves (ICC) for the questionnaire data collected one month post-operatively. (a) ICC for Glare bothersome; (b) ICC for Starbursts bothersome; (c) ICC for Haloes bothersome; (d) ICC for Blurred vision bothersome; (e) ICC for Hazy vision bothersome; (f) ICC for Double images bothersome; (g) ICC for Fluctuation bothersome; (h) ICC for Difficulty in depth perception bothersome; (i) ICC for Distortion bothersome.

From the estimates of symptoms’ locations in Table 6 and depicted in Fig 5, the most prevalent symptom within the cohort was Starbursts (ST), followed by Glare (GL), Blurred vision (BV), Haloes and Fluctuation (HL, FL), Hazy vision (HV) and Double images (DI), respectively; whereas the cohort under investigation was barely affected by Difficulty in depth perception (DDP) and Distortion (DS). These results are corroborated by the Item Characteristics Curves (ICCs) depicted in Fig 6, where the ICC for the response category “Not at all” dominates nearly all the ICCs for the other response categories for Distortion, and the ICCs of the response categories “Not at all” and “A little” dominate all the ICCs for the other response categories for Difficulty in depth perception. Furthermore, the ICCs suggest that the response category “Quite” is the least reported by this cohort of patients.

**Table 6:** Symptom location estimates (in logit), in terms of their level of prevalence within the cohort, and the corresponding standard errors, infits MNSQ and outfits MNSQ values, obtained from QoV questionnaire data collected one month post-operatively.

Symptom label	Symptom denomination	Symptom location (in logit)	Standard Error	Outfit MNSQ	Infit MNSQ
ST	Starbursts	-0.82	0.07	0.92	0.93
GL	Glare	-0.78	0.07	0.85	0.86
BV	Blurred vision	-0.50	0.08	0.97	0.99
HL, FL	Haloes, Fluctuation	-0.43	0.08	0.94	0.91
HV	Hazy vision	-0.13	0.09	0.84	0.97
DI	Double images	0.53	0.11	1.25	1.42
DDP	Difficulty in depth perception	0.85	0.13	1.19	1.38
DS	Distortion	1.69	0.19	1.05	1.45

The relatively high Outfit and/or Infit MNSQ values, compared to 1, for Group 2, Group 10, Group 16 and 17 indicated that most of the patients in these groups were annoyed by both the most and the least prevalent symptoms but not some of the other symptoms. However, this did not make these patients outliers.

The patients from this cohort who experienced most discomfort with their vision, and thus require additional care and monitoring, were those with higher location estimates. The top 10 patients, within the cohort, who experienced most discomfort with their vision are those in the rows highlighted in grey in Table 7, i.e. from Groups 12 to 17. From the questionnaire responses for these patients presented in Table 8, most of them reported significant discomfort from Glare (GL), Haloes (HL) and Starbursts (ST) but less from Distortion (DS) and Double images (DI) and to a certain extent Difficulty in depth perception (DDP). However, for the other symptoms their perception of visual discomfort is quite mixed.

**Table 7:** Patients’ location estimates (in logit), in terms of their perception of visual discomfort, and the corresponding standard errors, infit MNSQ and outfit MNSQ values, obtained from QoV questionnaire data collected one month post-operatively. The patient IDs, highlighted in bold, correspond to the top 10 patients with the most visual discomfort, one year post-operatively.

Group ID	Patients ID	Patient location (in logit)	Standard Error	Outfit MNSQ	Infit MNSQ	Percentage of patients per group
Group 1	1, 2, 3, 4, 19, 21, 28, 29, 33, 52, 56, 60, 65, 68, 69, 70, 76, 85, 91, 110, 114, 118, 122, 125, 132, 137, 142, 144, 150, 161, 164, 174, 175, 176, 179, 181, 182, 186, 188, 198, 199, 207, 214, 218, 231, 233, 236, 238, 242, 243, 252, 258, 267, 271, 276, 284, 285, 288, 297, 309, 313, 318, 322, 327, 336, 340, 351, 352, 354	-3.21	1.02	0.50	0.80	19.38%
Group 2	5, 15, 18, 31, 34, 35, 39, 40, 42, 44, 57, 58, 66, 71, 72, 74, 80, 81, 83, 87, 99, 106, 108, 112, **113**, 123, 131, 133, 134, 138, 152, 156, 158, 163, 168, 170, 171, 172, 187, 190, 195, 197, 205, **208**, 237, 239, 241, 253, 255, 256, 274, 279, 283, 296, 302, 311, 312, 314, 315, 317, 324, 329, 331, 332, 335, 337, 346, 350	-2.48	0.73	5.57	2.28	19.10%
Group 3	10, 11, 12, 16, 20, 23, 25, 36, 48, 54, 59, 64, 84, 100, 109, 115, 140, 143, 154, 160, 173, **180**, 185, 189, 196, 202, 213, 219, 240, 246, 248, 250, 260, 261, 268, 277, 278, 280, 282, 295, 298, 310, 319, 320, 323, 338, 339, 348	-2.04	0.61	0.64	0.76	13.48%
Group 4	17, 45, 47, 50, 55, 92, 93, 95, 98, 101, 102, 103, 105, 111, 116, 117, 135, 145, 146, 147, 149, **151**, 165, 169, 184, 191, 192, 193, 210, 220, 224, 225, 230, 234, 245, 249, 257, **262**, 265, 270, 275, 287, 290, 299, 306, 308, 321, 328, 334, 353, 355	-1.72	0.53	0.43	0.50	14.33%
Group 5	8, 13, 14, **24**, 26, 30, 37, 43, 46, 51, 78, 90, 97, 124, 129, 139, 183, 200, 201, 212, 226, 227, 244, 254, 272, **281**, 291, 301, 325, 326, 343, 344, 356	-1.46	0.49	0.25	0.24	9.27%
Group 6	38, 49, 73, 77, 82, 127, 159, **194**, 209, 215, 217, 222, 232, 235, 247, 264, 304, 305, 330	-1.24	0.45	0.74	0.83	5.62%
Group 7	6, 27, 41, 53, 89, 107, 136, 141, 177, 178, 221, 229, 251, 342	-1.04	0.43	1.25	0.81	3.93%
Group 8	22, 61, 75, 79, 86, 119, 120, 148, 153, 162, 216, **263**, 266, 289, 293, 303, 345, 349	-0.87	0.41	0.72	0.29	5.06%
Group 9	32, 62, 63, 67, 128, 203, **204**, 259, 273, 292, 333	-0.70	0.40	1.10	1.05	3.09%
Group 10	126, 157, 167, 286, 300, 341, 347	-0.54	0.39	2.49	1.48	1.97%
Group 11	96, 130, 155, 206, 211, 294, 307	-0.39	0.39	1.34	1.51	1.97%
Group 12	88, 223	-0.25	0.38	0.25	0.23	0.56%
Group 13	9, 94	-0.10	0.38	0.88	0.83	0.56%
Group 14	269	0.05	0.39	1.12	1.14	0.28%
Group 15	104, 228, 316	0.20	0.39	1.53	1.64	0.84%
Group 16	166	0.51	0.40	2.24	1.98	0.28%
Group 17	121	0.68	0.42	1.78	2.09	0.28%

**Table 8:** Questionnaire responses and locations (in logit) for the top 10 patients, who experienced most discomfort with their vision, identified by the Rasch model from QoV questionnaire data collected one month post-operatively.

Patients order	Patients ID	GL	HL	ST	HV	BV	DS	DI	FL	DDP	Location (in logit)
1^st^ Patient	121	Very	Very	Very	Not at all	Very	Not at all	Not at all	Very	Very	0.68
2^nd^ Patient	166	Very	Very	Not at all	Very	Very	Not at all	Not at all	Very	Quite	0.51
3^rd^ Patient	316	Very	Very	Very	Not at all	Very	Not at all	Not at all	Very	Not at All	0.20
4^th^ Patient	228	Very	Very	Very	Very	Quite	Not at all	Not at all	A little	Not at all	0.20
5^th^ Patient	104	Very	Very	Very	Very	Very	Not at all	Not at all	Not at all	Not at all	0.20
6^th^ Patient	269	Very	Not at all	Very	Quite	Quite	Not at all	Not at all	Quite	Quite	0.05
7^th^ Patient	94	Very	Quite	Very	Quite	Quite	Not at all	Not at all	A little	Not at all	-0.10
8^th^ Patient	9	A little	Very	Very	A little	Quite	A little	Not at all	Quite	Not at all	-0.10
9^th^ Patient	88	Quite	Quite	Quite	Quite	Quite	Not at all	A little	A little	Not at all	-0.25
10^th^ Patient	223	Very	Quite	Quite	Not at all	Very	Not at all	Not at all	Quite	Not at all	-0.25

#### Analysis of the questionnaire data collected one year post-operatively

**Fig 7:**
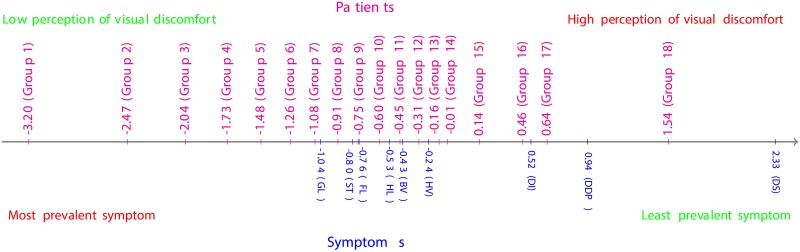
Patient-symptom map from QoV questionnaire data collected one year post-operatively.

**Fig 8:**
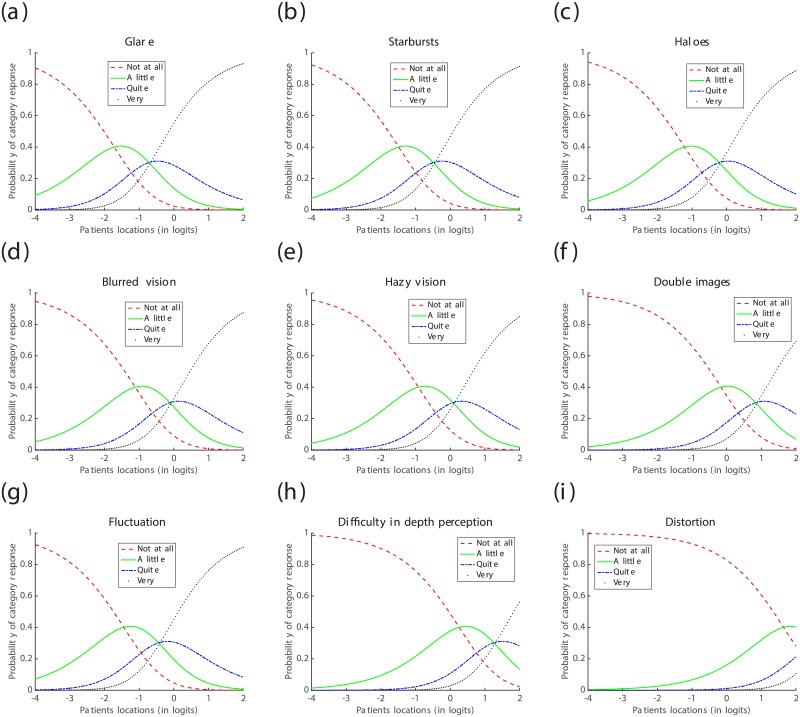
Item Characteristics Curves (ICC) for the questionnaire data collected one year post-operatively. (a) ICC for Glare bothersome; (b) ICC for Starbursts bothersome; (c) ICC for Haloes bothersome; (d) ICC for Blurred vision bothersome; (e) ICC for Hazy vision bothersome; (f) ICC for Double images bothersome; (g) ICC for Fluctuation bothersome; (h) ICC for Difficulty in depth perception bothersome; (i) ICC for Distortion bothersome.

From the estimates of the symptoms’ locations in Table 9 and depicted in Fig 7, the most prevalent symptom, within the cohort, was Glare (GL), followed by Starbursts (ST), Fluctuation (FL), Haloes (HL), Blurred vision (BV), Hazy vision (HV), Double images (DI) and Difficulty in depth perception (DDP), respectively; whereas the cohort under investigation was barely affected by Distortion (DS). These results are confirmed by the Item Characteristics Curves (ICCs) depicted in Fig 8, where the ICC for the response category “Not at all” dominates nearly all the ICCs for the other response categories for Distortion. Moreover, the ICCs suggest that the response category “Quite” was barely reported by the patients this time round. However, this does not provide enough ground to dismiss this response category. Only a continuous analysis of data collected from various cohorts of patients might enable the confirmation of an excessive subscaling of the response options, if any.

**Table 9:** Symptoms’ locations estimates (in logit), in terms of their level of prevalence within the cohort, and the corresponding standard errors, infits MNSQ and outfits MNSQ values, obtained from QoV questionnaire data collected one year post-operatively.

Symptom label	Symptom denomination	Symptom location (in logit)	Standard Error	Outfit MNSQ	Infit MNSQ
GL	Glare	-1.04	0.07	0.80	0.80
ST	Starbursts	-0.80	0.08	0.95	0.98
FL	Fluctuation	-0.76	0.08	0.98	0.97
HL	Haloes	-0.53	0.08	0.97	1.01
BV	Blurred vision	-0.43	0.09	0.94	0.98
HV	Hazy vision	-0.24	0.09	0.83	1.02
DI	Double images	0.52	0.12	1.30	1.57
DDP	Difficulty in depth perception	0.94	0.14	1.14	1.33
DS	Distortion	2.33	0.27	0.86	1.65

The relatively high Infit MNSQ values, compared to 1, for the symptom “Distortion”, indicated that this symptom affected patients who were the most and least annoyed with their vision, but this did not make this symptom irrelevant. The relatively high Outfit and/or Infit MNSQ values, compared to 1, for Group 4, Group 9, Group 11, Group 12, Group 15 and 16 indicated that most of the patients in these groups were most annoyed by both the most and the least prevalent symptoms but not some of the symptoms in between. However, this did not make these patients outliers.

One year post-operatively, the top 10 patients who were most annoyed with their vision are those in rows highlighted in grey in Table 10, i.e. from Groups 15 to 18. From the questionnaire responses, presented in Table 11, most of them reported significant discomfort from Glare (GL), Haloes (HL), Starbursts (ST), Blurred vision (BV), Hazy vision (HV) but not from Distortion (DS). Their perception of visual discomfort from the other symptoms is mixed.

**Table 10:** Patients’ location estimates (in logit), in terms of their perception of visual discomfort, and the corresponding standard errors, infit MNSQ and outfit MNSQ values, obtained from QoV questionnaire data collected one year post-operatively. The patient IDs, highlighted in bold, correspond to the top 10 patients with the most visual discomfort, one month post-operatively.

Group ID	Patients ID	Patient location (in logit)	Standard Error	Outfit MNSQ	Infit MNSQ	Percentage of patients per group
Group 1	1, 3, 10, 15, 16, 20, 22, 38, 48, 54, 57, 59, 61, 64, 65, 70, 77, 101, 103, 111, 116, 117, 124, 127, 128, 129, 130, 134, 135, 142, 146, 148, 150, 153, 158, 159, **166**, 167, 171, 173, 181, 182, 188, 190, 192, 196, 201, 202, 207, 215, 222, 225, 227, 230, 234, 245, 247, 248, 249, 252, 253, 265, 276, 278, 280, 284, 287, 294, 304, **316**, 318, 319	-3.20	1.02	0.42	0.74	22.50%
Group 2	7, 19, 23, 30, 33, 35, 37, 40, 41, 47, 53, 55, 60, 62, 74, **88**, 95, 97, 102, 118, 119, 123, 137, 141, 144, 145, 149, 161, 169, 175, 191, 218, 221, 226, **228**, 241, 242, 244, 254, 256, 259, 273, 277, 282, 288, 289, 290, 296, 300, 301, 305, 306, 308, 311, 315	-2.47	0.73	0.69	0.81	17.19%
Group 3	2, 12, 17, 34, 39, 42, 43, 45, 49, 52, 56, 67, 69, 80, **104**, 107, 108, 138, 152, 157, 162, 163, 164, 168, 172, 174, 185, 186, 189, 199, 214, 217, **223**, 232, 233, 243, 255, 257, 264, 266, 279, 285, 299, 302, 303, 310, 313, 314	-2.04	0.60	0.54	0.62	15.00%
Group 4	4, 11, 14, 18, 21, 44, 76, 81, 84, 85, 87, **94**, 96, 98, 120, 131, 132, 133, 136, 154, 170, 176, 177, 178, 193, 197, 206, 210, 213, 219, 237, 239, 246, 275, 292, 293, 317	-1.73	0.53	2.34	1.50	11.56%
Group 5	5, 8, 13, 26, 50, 73, 79, 89, 91, 99, 109, 115, 155, 183, 184, 187, 209, 212, 220, 250, 251, 260, 267, **269**, 286, 291, 307, 309	-1.48	0.48	0.67	0.36	8.75%
Group 6	6, **9**, 27, 28, 31, 32, 36, 66, 68, 71, 72, 100, 114, 156, 198, 203, 236, 240, 258, 271, 295	-1.26	0.44	0.68	0.73	6.56%
Group 7	25, 75, 83, 112, **121**, 160, 205, 224, 272, 320	-1.08	0.42	0.91	1.16	3.13%
Group 8	29, 46, 51, 58, 78, 90, 105, 122, 125, 179, 200, 216, 235, 238, 270, 274	-0.91	0.40	0.82	0.80	5.00%
Group 9	63, 82, 126, 165, 229, 261	-0.75	0.39	1.45	1.81	1.88%
Group 10	92, 106, 140, 143, 147, 211, 268, 297, 312	-0.60	0.39	0.63	0.66	2.81%
Group 11	110, 139, 231, 298	-0.45	0.38	1.60	1.87	1.25%
Group 12	86, 195	-0.31	0.38	2.76	2.47	0.63%
Group 13	93	-0.16	0.38	0.98	1.13	0.31%
Group 14	283	-0.01	0.39	0.41	0.39	0.31%
Group 15	113, 204, 281	0.14	0.39	2.12	2.37	0.94%
Group 16	24, 151, 180, 194	0.46	0.41	1.68	1.90	1.25%
Group 17	208, 262	0.64	0.43	0.81	1.03	0.63%
Group 18	263	1.54	0.54	1.05	0.88	0.31%

**Table 11:** Questionnaire responses and locations (in logit) for the top 10 patients, who experienced most discomfort with their vision identified by the Rasch model from QoV questionnaire data collected one year post-operatively.

Patients order	Patients ID	GL	HL	ST	HV	BV	DS	DI	FL	DDP	Location (in logit)
1^st^ Patient	263	Very	Very	Very	Very	Quite	Quite	Quite	Quite	Quite	1.54
2^nd^ Patient	262	Very	Very	Very	Quite	Very	Not at all	Not at all	Very	A little	0.64
3^rd^ Patient	208	Very	Very	Very	Very	Very	Not at all	Not at all	Very	Not at all	0.64
4^th^ Patient	194	Very	A little	A little	Very	Very	Not at all	Very	Very	Not at all	0.46
5^th^ Patient	180	Very	Very	Very	Very	Very	Not at all	Not at all	Quite	Not at all	0.46
6^th^ Patient	151	Very	Very	Not at all	Very	Very	Not at all	Not at all	Very	Quite	0.46
7^th^ Patient	24	Very	Not at all	Very	Very	Very	Not at all	Very	Quite	Not at all	0.46
8^th^ Patient	281	Very	Very	Very	Not at all	Very	Not at all	Not at all	Very	Not at all	0.14
9^th^ Patient	204	Very	Very	A little	Quite	Not at all	Not at all	A little	Quite	Very	0.14
10^th^ Patient	113	Very	Very	Very	Not at all	Very	Not at all	Very	Not at all	Not at all	0.14

#### Comparative analysis of the questionnaire data collected one month and one year post-operatively

**Fig 9:**
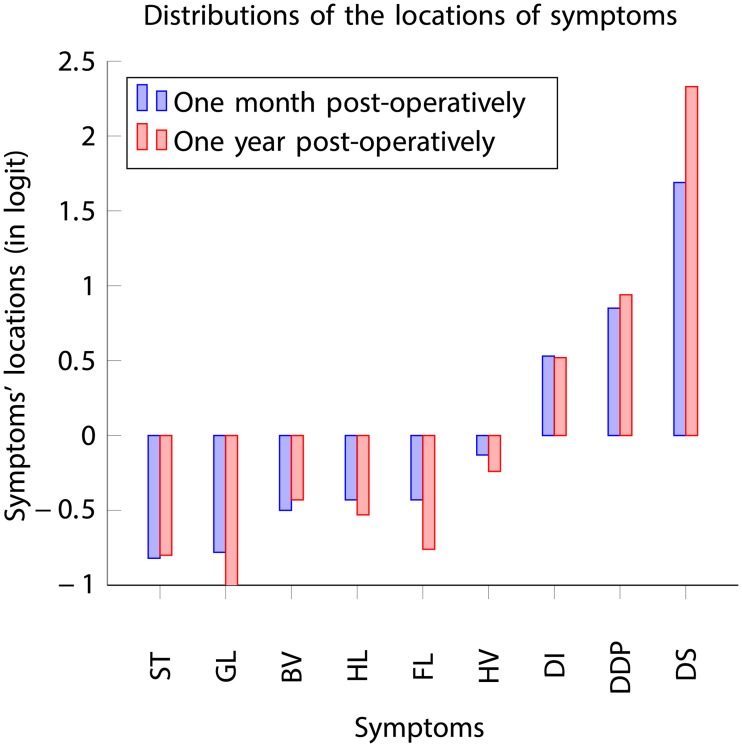
Location distributions of symptoms one month and one year post-operatively.

The distribution of the locations of symptoms (in logit), depicted in Fig 9, showed a noticeable decrease in the prevalence of the symptom Distortion (DS) and a slight decrease in the prevalence of the symptoms Difficulty in depth perception (DDP) and Blurred vision (BV) one year post-operatively, while an increase in the prevalence of the symptoms Glare (GL), Fluctuation (FL) and Haloes (HL) are observed within the overall cohort of patients.

**Fig 10:**
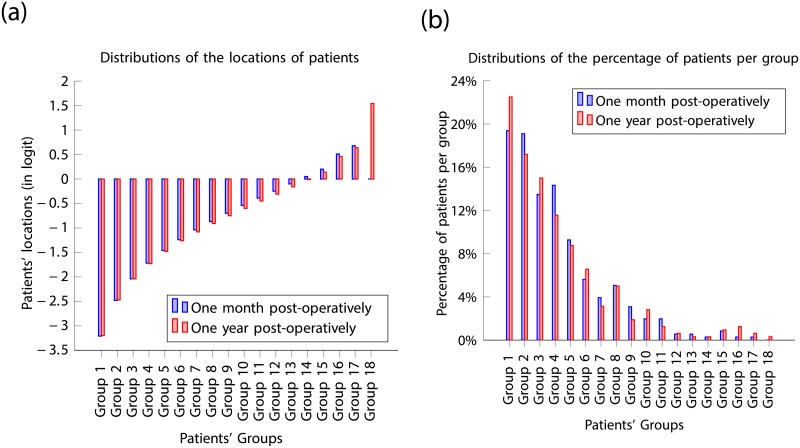
(a) Location distributions of patients one month and one year post-operatively; (b) Distributions of patients percentage per group one month and one year post-operatively.

The distribution of the locations of patients (in logit), in Fig 10(a), showed globally little variation in terms of the level of perception of visual discomfort within the cohort one month and one year post operatively. However, the results point-out that Patient 263 (Group 18) was significantly annoyed with his/her vision, one year post-operatively which was not the case one month post-operatively. From the distribution of patients per group, in Fig 10(b), there was a relative increase in both the fractions of patients who experienced less and more visual discomfort one year post-operatively compared to eleven months earlier.

**Fig 11:**
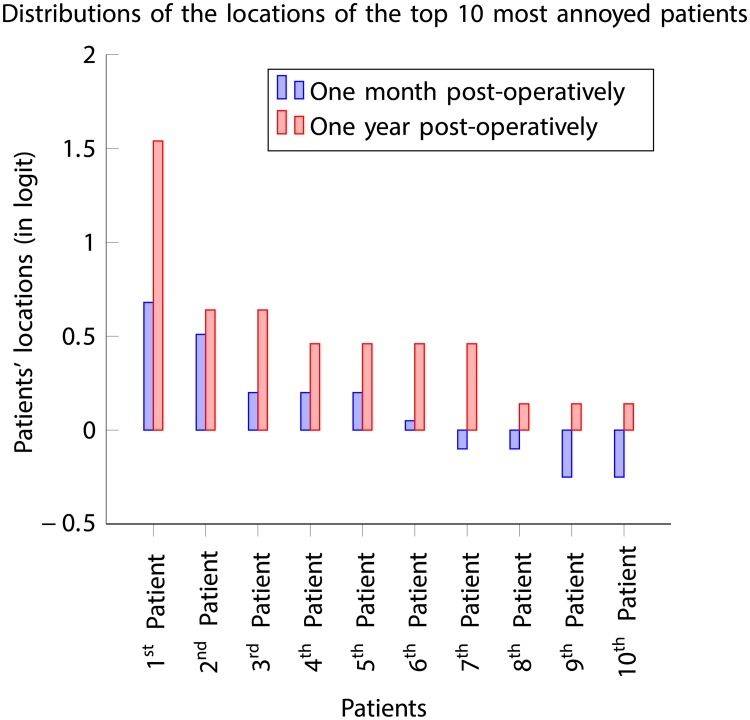
Location distributions of the top 10 patients, who were most annoyed with their vision, one month and one year post-operatively.

The cohort of the top 10 patients, who were most annoyed with their vision one month post-operatively (Table 8) is entirely different from the cohort of the top 10 who experienced most discomfort one year post-operatively (Table 11). The top 10 patients, who were most annoyed with their vision one-month post-operatively, highlighted in bold in Table 10, have shown a significant improvement in their perception of visual discomfort. On the other hand, the top 10 patients who were most annoyed with their vision one year post-operatively, highlighted in bold in Table 7, were generally mildly annoyed with their vision one month post-operatively. The location distribution results, depicted in Fig 11, showed that the level of perception of visual discomfort from the top 10 patients is substantially higher one year post-operatively compared to one month post-operatively.

**Remark 3** *Following the approach advocated in previous studies, e.g.* [10]–[6], [29]–[7], [8]–[30], *which use the misfit statistics of the items and the Items Characteristics Curves (ICCs), to dismiss* [9]–[18] *or approve* [9], [14], [21]–[30] *an ophthalmic questionnaire, the following conclusions can be drawn on the QoV questionnaire used in this study: one month post-operatively, the values of the Outfit MNSQ and Infit MNSQ statistics, for all the symptoms, were below the 1.5 threshold and were not far away from the expected value of 1; all the response categories were expressed in the ICCs of most of the symptoms, except for Double Images (DI), Distortion (DS) and Difficulty in depth perception (DDP); hence, the QoV questionnaire is “Rasch-valid” after the removal of the aforementioned three symptoms*.

*On the other hand, the same questionnaire, administered to the same cohort of patients, becomes “Rasch-invalid” eleven months later, i.e. one year post-operatively, since the category response “Quite” was no longer expressed in the ICCs of all the symptoms and the values of the Infit MNSQ statistics for the symptoms Double images (DI) and Distortion (DS) exceeded the 1.5 threshold*.

*These findings shed light on some of the major flaws associated with the current most advocated approach for validating ophthalmic questionnaires using Rasch analysis, e.g.* [10]–[6], [29]–[7], [8]–[30], *which cast some serious doubt about its “validity”*.

## 4 Alternative approach in the application of Rasch model to assess ophthalmic PROs data

Unlike the current applications of the Rasch model to validate ophthalmic questionnaires, the alternative application of Rasch analysis, proposed in this study, enables a meaningful use of Rasch analysis as an intelligent decision support system for deriving valuable insights from data collected via ophthalmic questionnaires. At the population level, such an approach enables one to investigate the prevalence of ophthalmic symptoms across different cohorts of patients, through a better characterization of patient groups pre-operatively and an appropriate follow-up post-operatively, in order to assess the effectiveness of a treatment—e.g. different types of intraocular lenses (IOLs) or different surgical procedures. At the individual level, the new approach can be applied across a population at different time points and identify patients who experienced most visual discomfort pre-operatively and/or post-operatively, so that additional appropriate care and monitoring can be dedicated to them. This new perspective will pave the way for a more adequate application of Rasch analysis within the context of ophthalmic questionnaires, so that insights gained from the analysis can be exploited to enhance the quality of care and patient care experience.

For illustrative purposes, the new approach was used to investigate the prevalence of QoV related symptoms across a cohort of patients at different time points. The analysis of the questionnaire data, using the new approach in the application of Rasch model, was used to characterize the variation in the prevalence of symptoms, from one month to one year post-operatively, and to identify the patients who experience the most visual discomfort at these two time points, and therefore can receive additional care and monitoring.

The purpose of this paper was not to attempt to advocate an alternative validation method of ophthalmic questionnaires or to supersede Rasch analysis but to highlight the importance of continuous assessment and monitoring of questionnaire data through Rasch analysis instead of simply dismissing or approving questionnaires based on a study of single cohort at a given, and present Rasch analysis as a decision support tool for deriving insights from data obtained using ophthalmic questionnaires. We will use the proposed alternative application of Rasch analysis to assess and compare the effectiveness of various IOLs, and to investigate the impact of patient characteristics such as lifestyle, age and gender, on the perception of visual discomfort post-operatively. Our future work will also further investigate validation methods of ophthalmic questionnaires.

## A Appendix A—Derivation of the dichotomous Rasch model

If the responses to test items consist of only two categories then dichotomous item response models can be applied. Without loss of generality, we can assume that the response of any examinee *p* to any item *i*, *u*_*pi*_, can only be either 0 or 1. From Assumption 3, the response of an examinee *p* to item *i*, *u*_*pi*_, depends on a single parameter *ξ*_*pi*_, which goes from 0 to ∞. Thus, the response probability for an examinee *p* to an item *i* can be defined by any continuous and monotonic function of *ξ*_*pi*_, which takes on only the values from 0 to 1, as *ξ*_*pi*_ goes from 0 to ∞. Rasch suggested [1, 3] the following simple function:

$$\begin{array}{c} f\left(\xi_{pi}\right)=\frac{\xi_{pi}}{1+\xi_{pi}}. \end{array}$$ (6)

Therefore,

$$P(u_{pi}=1|\xi_{pi})=\frac{\xi_{pi}}{1+\xi_{pi}},\text{and}$$ (7)

$$P(u_{pi}=0|\xi_{pi})=1-\frac{\xi_{pi}}{1+\xi_{pi}}=\frac{1}{1+\xi_{pi}}.$$ (8)

Eqs (7) and (8) can then be written in a general form, as follows

$$\begin{array}{c} P\left(u_{pi}|\xi_{pi}\right)=\frac{{\left(\xi_{pi}\right)}^{u_{pi}}}{1+\xi_{pi}}. \end{array}$$ (9)

Substituting *ξ*_*pi*_ by $\frac{a_{p}}{d_{i}}$ in Eq (9) yields

$$\begin{array}{c} P(u_{pi}|a_{p},d_{i})=\frac{{\left(\frac{a_{p}}{d_{i}}\right)}^{u_{pi}}}{1+\frac{a_{p}}{d_{i}}}. \end{array}$$ (10)

However, the above formulation restricted the parameter *ξ*_*pi*_ to vary from 0 to ∞. Since, $\xi_{pi}=\frac{a_{p}}{d_{i}}$, then this formulation restricted the ability and the difficulty parameters, *a*_*p*_ and *d*_*i*_, respectively, to be either both positive or negative. However, it would be preferable to have a formulation where both the ability and the difficulty parameters can be used irrespective of their signs. One way to address the limitation of the above formulation is to consider a logarithmic transformation of both the ability and difficulty parameters as follows:

$${\hat{a}}_{p}=\text{log}(a_{p})$$ (11)

$${\hat{d}}_{i}=\text{log}(d_{i}).$$ (12)

Now, the rescaled ability and difficulty parameters ${\hat{a}}_{p}$ and ${\hat{d}}_{i}$, respectively vary from −∞ to +∞, and the following inverse transformation enables the recovery of the initial ability and difficulty parameters *a*_*p*_ and *d*_*i*_:

$$a_{p}=e^{{\hat{a}}_{p}}$$ (13)

$$d_{i}=e^{{\hat{d}}_{i}}.$$ (14)

Substituting *a*_*p*_ and *d*_*i*_ by $e^{{\hat{a}}_{p}}$ and $e^{{\hat{d}}_{i}}$ respectively, in Eq (10) yields

$$P(u_{pi}|{\hat{a}}_{p},{\hat{d}}_{i})=\frac{{\left(e^{{\hat{a}}_{p}}e^{-{\hat{d}}_{i}}\right)}^{u_{pi}}}{1+e^{{\hat{a}}_{p}}e^{-{\hat{d}}_{i}}}=\frac{e^{\left({\hat{a}}_{p}-{\hat{d}}_{i}\right)u_{pi}}}{1+e^{\left({\hat{a}}_{p}-{\hat{d}}_{i}\right)}}.$$ (15)

Thus,

$$P(u_{pi}=1|{\hat{a}}_{p},{\hat{d}}_{i})=\frac{e^{\left({\hat{a}}_{p}-{\hat{d}}_{i}\right)}}{1+e^{\left({\hat{a}}_{p}-{\hat{d}}_{i}\right)}}=\frac{1}{1+e^{-({\hat{a}}_{p}-{\hat{d}}_{i})}},\text{and}$$ (16)

$$P(u_{pi}=0|{\hat{a}}_{p},{\hat{d}}_{i})=\frac{1}{1+e^{\left({\hat{a}}_{p}-{\hat{d}}_{i}\right)}}.$$ (17)

### A.1 Some mathematical properties of the Rasch model

#### Linearity

The extension of the logarithmic transformation (11)-(12) to the parameter *ξ*_*pi*_ leads to the following result:

$$\begin{array}{c} \text{log}\left(\xi_{pi}\right)=\text{log}(\frac{a_{p}}{d_{i}})=\text{log}\left(a_{p}\right)-\text{log}\left(d_{i}\right)=\text{log}\left(e^{{\hat{a}}_{p}}\right)-\text{log}\left(e^{{\hat{d}}_{i}}\right)={\hat{a}}_{p}-{\hat{d}}_{i}. \end{array}$$ (18)

Hence, after the above logarithmic transformation, the response probability of an examinee *p* to an item *i* is governed by the difference between ${\hat{a}}_{p}$ and ${\hat{d}}_{i}$. In other words, the response probability depends only on the distance between the examinee’s ability and the item difficulty parameters both on the logit scale, i.e. a line similar to the one described in Fig 2. Therefore, the derived model becomes an additive model.

#### Separation of parameters

Assumption 3 and Assumption 4 confer the Rasch model some desirable mathematical features, which enable the estimation of the two classes of parameters of the model, i.e. ${\hat{a}}_{p}$ and ${\hat{d}}_{i}$, from the data response matrix, independently from one another. Given the Rasch model (15) and its parameters ${\hat{a}}_{p}$ and ${\hat{d}}_{i}$, which are not known yet, and a response data matrix *U*, the probability of the whole response data matrix—i.e. the likelihood, denoted *L*—consists of the following continued product.

$$\begin{array}{c} \begin{array}{cc} L=P(u_{pi}|{\hat{a}}_{p},{\hat{d}}_{i})= & \prod_{p=1}^{m}\prod_{i=1}^{n}\frac{e^{\left({\hat{a}}_{p}-{\hat{d}}_{i}\right)u_{pi}}}{1+e^{\left({\hat{a}}_{p}-{\hat{d}}_{i}\right)}}\\ = & \frac{\prod_{p=1}^{m}\prod_{i=1}^{n}e^{\left({\hat{a}}_{p}-{\hat{d}}_{i}\right)u_{pi}}}{\prod_{p=1}^{m}\prod_{i=1}^{n}(1+e^{\left({\hat{a}}_{p}-{\hat{d}}_{i}\right)})}\\ = & \frac{e^{\sum_{p=1}^{m}\sum_{i=1}^{n}\left({\hat{a}}_{p}-{\hat{d}}_{i}\right)u_{pi}}}{\prod_{p=1}^{m}\prod_{i=1}^{n}(1+e^{\left({\hat{a}}_{p}-{\hat{d}}_{i}\right)})}\\ = & \frac{e^{\sum_{p=1}^{m}{\hat{a}}_{p}\sum_{i=1}^{n}u_{pi}}\times e^{-\sum_{i=1}^{n}{\hat{d}}_{i}\sum_{p=1}^{m}u_{pi}}}{\prod_{p=1}^{m}\prod_{i=1}^{n}(1+e^{\left({\hat{a}}_{p}-{\hat{d}}_{i}\right)})}. \end{array} \end{array}$$ (19)

The most desirable parameters ${\hat{a}}_{p}$ and ${\hat{d}}_{i}$ for the Rasch model are those such that the likelihood, *L*, is maximal. However, obtaining these parameters from (19) can be tedious due to the complexity of the expression of likelihood *L*. On the other hand, the parameters ${\hat{a}}_{p}$ and ${\hat{d}}_{i}$, which maximize *L* are identical to those which maximize the logarithm of *L*. The logarithm of *L*, i.e. the log likelihood, of the data matrix *U*, writes

$$\begin{array}{c} \Gamma =\text{log}L=\sum_{p=1}^{m}{\hat{a}}_{p}s_{p}-\sum_{i=1}^{n}{\hat{d}}_{i}s_{i}-\sum_{p=1}^{m}\sum_{i=1}^{n}\text{log}(1+e^{\left({\hat{a}}_{p}-{\hat{d}}_{i}\right)}), \end{array}$$ (20)

where $s_{p}=\sum_{i=1}^{n}u_{pi}$ and $s_{i}=\sum_{p=1}^{m}u_{pi}$ denote the total score of the examinee *p* and the item *i*, respectively.

In order to estimate the desirable parameters ${\hat{a}}_{p}$ and ${\hat{d}}_{i}$, we need to solve the system (21)-(22), and the corresponding solution needs to satisfy the conditions (23)-(24).

$$\frac{\partial \Gamma }{\partial {\hat{a}}_{p}}=s_{p}-\sum_{i=1}^{n}\gamma_{pi}=0,$$ (21)

$$\frac{\partial \Gamma }{\partial {\hat{d}}_{i}}=-s_{i}-\sum_{p=1}^{m}\gamma_{pi}=0;$$ (22)

$$\frac{\partial \Gamma }{\partial {\hat{a}}_{p}}=-\sum_{i=1}^{n}\gamma_{pi}\left(1-\gamma_{pi}\right)\leq 0,$$ (23)

$$\frac{\partial \Gamma }{\partial {\hat{d}}_{i}}=-\sum_{p=1}^{m}\gamma_{pi}\left(1-\gamma_{pi}\right)\leq 0;$$ (24)

where $\gamma_{pi}=\frac{e^{\left({\hat{a}}_{p}-{\hat{d}}_{i}\right)}}{1+e^{\left({\hat{a}}_{p}-{\hat{d}}_{i}\right)}}$.

An additional condition, namely $\sum_{i=1}^{n}{\hat{d}}_{i}=0$, is included to the system (21) in order to have the item parameters ${\hat{d}}_{i}$ centered at zero. It is worth mentioning that the parameters obtained from (21) and (23) are not deficiency free. Indeed, these estimates assume that the person score *s*_*p*_ is independent from the difficulty of the items in the test, and likewise the item score *s*_*i*_ is independent from the ability distribution of the persons tested. However, none of these assumptions are generally satisfied in practice. An adjustment of the observed scores *s*_*p*_ and *s*_*i*_ to the corresponding item difficulty and person ability distributions are required to estimate the desirable test-free person parameters ${\hat{a}}_{p}$ and sample-free item parameters ${\hat{d}}_{i}$ [31].

## B Appendix B—Derivation of the misfit statistics for the Rasch model

For the dichotomous Rasch model, the response of person *p* to an item *i*, *u*_*pi*_, is a variable following a Bernoulli distribution, i.e. it takes only two values, e.g. 0 and 1. The Rasch model estimates the probability of any instance of response *u*_*pi*_ as

$$\begin{array}{c} P\left(u_{pi}|{\hat{a}}_{p},{\hat{d}}_{i}\right)=\frac{e^{\left({\hat{a}}_{p}-{\hat{d}}_{i}\right)u_{pi}}}{1+e^{\left({\hat{a}}_{p}-{\hat{d}}_{i}\right)}}, \end{array}$$

where ${\hat{a}}_{p}$ is the estimated ability parameter of the person *p* and ${\hat{d}}_{i}$ is the estimated difficulty parameter of item *i*.

The expected value of instances of *u*_*pi*_, denoted ${\hat{u}}_{pi}$, is given by

$$\begin{array}{c} \begin{array}{cc} {\hat{u}}_{pi} & =E(u_{pi})\\ & =P\left(u_{pi}=0|{\hat{a}}_{p},{\hat{d}}_{i}\right)\times 0+P\left(u_{pi}=1|{\hat{a}}_{p},{\hat{d}}_{i}\right)\times 1\\ & =\frac{e^{\left({\hat{a}}_{p}-{\hat{d}}_{i}\right)}}{1+e^{\left({\hat{a}}_{p}-{\hat{d}}_{i}\right)}}. \end{array} \end{array}$$ (25)

The variance of instances of *u*_*pi*_ is given by

$$\begin{array}{c} \begin{array}{cc} \text{Var}(u_{pi}) & =E\left(u_{pi}^{2}\right)-(E{\left(u_{pi}\right)}^{2}\\ & =P\left(u_{pi}^{2}=0|{\hat{a}}_{p},{\hat{d}}_{i}\right)\times 0+P\left(u_{pi}^{2}=1|{\hat{a}}_{p},{\hat{d}}_{i}\right)\times 1-{\hat{u}}_{pi}^{2}\\ & ={\hat{u}}_{pi}\left(1-{\hat{u}}_{pi}\right). \end{array} \end{array}$$ (26)

The residual, i.e. the difference between the observed value of *u*_*pi*_ and its estimated value ${\hat{u}}_{pi}$, obtained via the Rasch model, is given by

$$\begin{array}{c} r_{pi}=u_{pi}-{\hat{u}}_{pi}. \end{array}$$

The standard residual, i.e. the residual divided by the expected standard deviation of instances of *u*_*pi*_ obtained from (26), is given by

$$\begin{array}{c} \begin{array}{cc} z_{pi} & =\frac{r_{pi}}{\sqrt{{\hat{u}}_{pi}\left(1-{\hat{u}}_{pi}\right)}}\\ & =\frac{u_{pi}-{\hat{u}}_{pi}}{\sqrt{{\hat{u}}_{pi}\left(1-{\hat{u}}_{pi}\right)}}. \end{array} \end{array}$$ (27)

The expected value of the standard residuals, denoted ${\hat{z}}_{pi}$, is given by

$$\begin{array}{c} \begin{array}{cc} {\hat{z}}_{pi} & =E(z_{pi})\\ & =E(\frac{u_{pi}-{\hat{u}}_{pi}}{\sqrt{{\hat{u}}_{pi}\left(1-{\hat{u}}_{pi}\right)}})\\ & =\frac{E\left(u_{pi}\right)-{\hat{u}}_{pi}}{\sqrt{{\hat{u}}_{pi}\left(1-{\hat{u}}_{pi}\right)}}\\ & =0. \end{array} \end{array}$$ (28)

The variance of the standard residuals is given by

$$\begin{array}{c} \begin{array}{cc} \text{Var}(z_{pi}) & =\text{Var}(\frac{u_{pi}-{\hat{u}}_{pi}}{\sqrt{{\hat{u}}_{pi}\left(1-{\hat{u}}_{pi}\right)}})\\ & =\frac{\text{Var}(u_{pi})}{{\hat{u}}_{pi}\left(1-{\hat{u}}_{pi}\right)}\\ & =\frac{{\hat{u}}_{pi}\left(1-{\hat{u}}_{pi}\right)}{{\hat{u}}_{pi}\left(1-{\hat{u}}_{pi}\right)}\\ & =1. \end{array} \end{array}$$ (29)

Therefore, the standard deviation of the standard residuals, *z*_*pi*_, is 1.

For a large response data matrix, the standard residuals approximate a standard normal distribution with a mean of 0 and a standard deviation of 1, i.e.

$$\begin{array}{c} z_{pi}∼N\left(0,1\right), \end{array}$$

and consequently, the square of standard residuals approximate a chi-square distribution with one degree of freedom, i.e.

$$\begin{array}{c} z_{pi}^{2}∼\chi_{1}^{2}. \end{array}$$

Either of the above reference distributions, i.e. N(0,1) and $\chi_{1}^{2}$, can be used to assess the significance of the deviation of the standard residuals from their expected values. On the one hand, the analysis of the standard residuals enables the identification of ill-defined items, if any, which require further refinement to be inline with reasonable expectations. Furthermore, the standard residuals enable the identification of persons, if any, whose responses deviated from reasonable expectations [31].

### B.1 Item misfit statistics

The infit mean square statistic for item *i*, denoted Infit MNSQ_*i*_, is given by the following weighted sum of the mean square residuals:

$$\begin{array}{c} \text{Infit}\;{\text{MNSQ}}_{i}=\frac{\sum_{p=1}^{m}\text{Var}\left(u_{pi}\right)z_{pi}^{2}}{\sum_{p=1}^{m}\text{Var}\left(u_{pi}\right)}. \end{array}$$

The outfit mean square statistic for item *i*, denoted Outfit MNSQ_*i*_, is given by the unweighted sum of the mean square residuals:

$$\begin{array}{c} \text{Outfit}\;{\text{MNSQ}}_{i}=\frac{\sum_{p=1}^{m}z_{pi}^{2}}{m}. \end{array}$$

Although some of the statistical properties of the above outfit and infit statistics are not fully known, they are generally assumed to approximate a standard normal distribution (i.e. with a mean of 0 and a standard deviation of 1) in Rasch analysis literature. However, the distribution of their following cube-root transformation, suggested by Wilson and Hilferty [38], approximate a scaled chi-squared distributions. The transformed outfit and infit statistics are referred to as the outfit *z*-standardized and the intfit *z*-standardized, respectively, in Rasch analysis literature.

The intfit *z*-standardized statistics for item *i*, denoted Intfit ZSTD_*i*_, is given by

$$\begin{array}{c} \text{Infit}\;{\text{ZSTD}}_{i}=\frac{3(k_{i}^{1/3}-1)}{q_{i}}+\frac{q_{i}}{3}, \end{array}$$

where *k*_*i*_ = Infit MNSQ_*i*_ and *q*_*i*_ is the standard deviation of the infit mean square statistic for item *i*.

The outfit *z*-standardized statistics for item *i*, denoted Outfit ZSTD_*i*_, is given by

$$\begin{array}{c} \text{Outfit}\;{\text{ZSTD}}_{i}=\frac{3({\hat{k}}_{i}^{1/3}-1)}{{\hat{q}}_{i}}+\frac{{\hat{q}}_{i}}{3}, \end{array}$$

where ${\hat{k}}_{i}=\text{Outfit}\;{\text{MNSQ}}_{i}$ and ${\hat{q}}_{i}$ is the standard deviation of the outfit mean square statistic for item *i*.

### B.2 Person misfit statistics

Like for items, the mean square misfit statistics for a person *p* are given by:

$$\begin{array}{cc} & \text{Infit}\;{\text{MNSQ}}_{p}=\frac{\sum_{i=1}^{n}\text{Var}\left(u_{pi}\right)z_{pi}^{2}}{\sum_{i=1}^{n}\text{Var}\left(u_{pi}\right)}, \end{array}$$ (30)

$$\begin{array}{cc} & \text{Outfit}\;{\text{MNSQ}}_{p}=\frac{\sum_{i=1}^{n}z_{pi}^{2}}{n}. \end{array}$$ (31)

The *z*-standardized misfit statistics for a person *p* are given by:

$$\begin{array}{cc} & \text{Infit}\;{\text{ZSTD}}_{p}=\frac{3(k_{p}^{1/3}-1)}{q_{p}}+\frac{q_{p}}{3}, \end{array}$$ (32)

$$\begin{array}{cc} & \text{Outfit}\;{\text{ZSTD}}_{p}=\frac{3({\hat{k}}_{p}^{1/3}-1)}{{\hat{q}}_{p}}+\frac{{\hat{q}}_{p}}{3}, \end{array}$$ (33)

with *k*_*p*_ = Infit MNSQ_*p*_, ${\hat{k}}_{p}=\text{Outfit}\;{\text{MNSQ}}_{p}$, whereas *q*_*p*_ and ${\hat{q}}_{i}$ are the standard deviations of the infit mean square and the outfit mean square statistic for person *p*, respectively.

## Supporting information

S1 Dataset One month post operative data. This file contains the questionnaire data collected one month post-operatively. The column names indicate the 9 symptoms corresponding to the items. The rows correspond to the patients. The value 1, 2, 3 and 4 correspond to the severity levels “Not at all”, “A little”, “Quite”, and “Very”, respectively.
(XLSX)

S2 Dataset One year post operative data. This file contains the questionnaire data collected one year post-operatively. The column names indicate the 9 symptoms corresponding to the items. The rows correspond to the patients. The value 1, 2, 3 and 4 correspond to the severity levels “Not at all”, “A little”, “Quite”, and “Very”, respectively.
(XLSX)

S1 Script This file contained the program of the Rash model for polytomous item response, where the estimation of the parameters is based on the joint maximum likelihood estimation (JMLE). (P)

S2 Script This file contains the main Matlab script which should be run on Matlab 2014b or most recent version. When running this program, all the files (namely main_script.m, fct_poly_rasch_analysis.p, One_Month_Post_Operative_Data.xlsx, One_Year_Post_Operative_Data.xlsx) should be in current working directory. After execution, following the comments on the top of the file, the program produced the results in Tables 6, 7, Fig 6, Tables 9, 10 and Fig 8 in the manuscript, respectively.
(M) Click here for additional data file.

S1 Fig This file contains a sample of images used when answering questions for the QoV questionnaire. (EPS)
